# Measures of Coupling between Neural Populations Based on Granger Causality Principle

**DOI:** 10.3389/fncom.2016.00114

**Published:** 2016-10-26

**Authors:** Maciej Kaminski, Aneta Brzezicka, Jan Kaminski, Katarzyna J. Blinowska

**Affiliations:** ^1^Department of Biomedical Physics, Faculty of Physics, University of WarsawWarsaw, Poland; ^2^Department of Psychology, University of Social Sciences and HumanitiesWarsaw, Poland; ^3^Centre for Modern Interdisciplinary Technologies, Nicolaus Copernicus UniversityTorun, Poland; ^4^Institute of Biocybernetics and Biomedical Engineering of Polish Academy of SciencesWarsaw, Poland

**Keywords:** neural synchronization, Granger causality, Directed Transfer Function, effective connectivity, causal coupling

## Abstract

This paper shortly reviews the measures used to estimate neural synchronization in experimental settings. Our focus is on multivariate measures of dependence based on the Granger causality (G-causality) principle, their applications and performance in respect of robustness to noise, volume conduction, common driving, and presence of a “weak node.” Application of G-causality measures to EEG, intracranial signals and fMRI time series is addressed. G-causality based measures defined in the frequency domain allow the synchronization between neural populations and the directed propagation of their electrical activity to be determined. The time-varying G-causality based measure Short-time Directed Transfer Function (SDTF) supplies information on the dynamics of synchronization and the organization of neural networks. Inspection of effective connectivity patterns indicates a modular structure of neural networks, with a stronger coupling within modules than between them. The hypothetical plausible mechanism of information processing, suggested by the identified synchronization patterns, is communication between tightly coupled modules intermitted by sparser interactions providing synchronization of distant structures.

## Introduction

In recent years a substantial effort has been directed toward elucidating the role of synchronization in mechanisms of neural population coupling. The kind of measure applied to estimate connectivity patterns plays a crucial role in the understanding of this synchronization. A multitude of methods have been devised for estimation of connectivity between neural populations: linear and non-linear, bivariate and multivariate, directed and undirected. It is impossible to describe all of the measures of synchronization in this mini review, but they are described in the review by Blinowska (2011) and in the book by Blinowska and Zygierewicz (2011). The effectiveness of causality measures was compared e.g., by Astolfi et al. (2007), Schlögl and Supp (2006), and Dauwels et al. (2010). Here we will focus on the methods that, going beyond the statistical assessment of synchronization, provide information on causal coupling.

## Connectivity measures

Among the most frequently used connectivity measures defined in the time domain, namely cross-correlation, Mutual Information, Transfer Entropy (TE) and Granger Causality Index (GCI), the last two indicate the directedness of information flow. For time-metric methods a contribution of different rhythms may be estimated by means of filtering; however, methods operating in the frequency domain are more convenient for synchronization assessment. In the frequency domain we can distinguish several measures of functional connectivity: coherence, pair-wise measures based on phase information such as the phase lag index (Stam et al., 2007), and pairwise phase consistency (Vinck et al., 2011). Functional connectivity is only a statistical measure of interdependence—phase difference does not imply a causal relation between signals of interest. Another problem connected with the above mentioned bivariate measures of functional connectivity is the generation of spurious connections, which was demonstrated in Kus et al. (2004) and Blinowska et al. (2004). Effective connectivity indicates a causal relation, providing information on the influence exerted by a given channel on other channels. Effective connectivity measures, i.e., Granger Causality (GC), Directed Transfer Function (DTF) (Kamiński and Blinowska, 1991) and Partial Directed Coherence (PDC) (Baccala and Sameshima, 2001), are based on the Granger's causality (G-causality) principle. They are extensions to a multivariate case of Granger's original idea concerning two signals (Granger, 1969). When assessing the usefulness of a method, the following features should be taken into account: robustness in respect to noise and volume conduction and influence of the common feeding effect.

The common input problem is a source of a serious pitfalls, corrupting all bivariate measures and leading to the creation of spurious connections. Namely, if signals propagating from a given source are measured at *N* electrodes, bivariate methods may show not just *N* active connections between source and sensors, but instead, by virtue of common feeding, it will be *N* (*N* − 1)/2. Therefore, one may get more false than true connections. In consequence, many papers based on bivariate methods report very dense and almost random connectivity patterns (Blinowska and Kaminski, 2013).

Volume conduction—a factor limiting the spatial resolution of synchronization measures—is connected to propagation of the electromagnetic field. Since the electromagnetic field propagates at the speed of light, it does not produce phase differences on the electrodes; hence, methods based on phase differences (among them DTF and PDC) are hardly influenced by volume conduction (Kaminski and Blinowska, 2014). Causal information is coded in the delays between given signals. Pre-processing such as the Hjorth transform or projection into source space involves mathematical operations that mix the information from the signals of the set, so the phase information is lost. Therefore, this kind of pre-processing should be avoided.

Non-linear methods of connectivity are much more affected by noise than the linear ones. Moreover, they are prone to systematic errors (Pereda et al., 2005; Netoff et al., 2006). According to some authors, application of non-linear methods is recommended only when strong evidence of non-linearity is present. Surrogate data tests and linear-vs. non-linear forecasting indicate that non-linearity in EEG and LFP (Local Field Potentials) is rather exceptional, and practically appears only in some phases of epileptic seizure (Blinowska and Malinowski, 1991; Achermann et al., 1994; Pijn et al., 1997; Stam et al., 1999). In fact, G-causality based measures perform quite well for non-linear signals. It has been established (Barnett et al., 2009) that for Gaussian variables, the non-linear measure TE is equivalent to GC; however, computations are easier and more reliable for GC.

## Multivariate G-causality measures

The notion of causality in time series, based on Wiener's idea (Wiener, 1956), was introduced by Granger (1969). In general, Granger Causality (GC) represents the improvement of predicting values of signal *X* when not only the previous history of *X* but also previous history of another signal *Y* is taken into account. The measure is expressed as a log value of prediction accuracy ratios in both cases. This measure may be conditioned on other signals. The GC was further developed by Geweke (1982, 1984) in both—time and frequency—domains. In practice, it was found that Geweke's frequency-domain conditional G-causality measure generates negative values in certain cases. This unfortunate property leads to the definition of a modified conditional G-causality based on the partition matrix technique (Chen et al., 2006).

In the identification of casual relations one should try to incorporate all possible variables of the process. However, that may be difficult because of the influence of exogenous (environmental) and latent (unmeasured) variables. The problem of eliminating these confounding inputs was confronted by Eichler (2005) who proposed a graphical approach. In Guo et al. (2008) the partial G-causality measure was introduced. This method was tested by simulations and application to multichannel LFP.

GC was successfully used e.g., to evaluate the directional influences in large-scale sensorimotor cortical networks (Brovelli et al., 2004) and oscillatory synchronization in top-down neocortical processing (Bastos and Schoffelen, 2016). Some additional applications of GC in the study of the nervous system, latent variable control, and relations with Dynamic Causal Modeling are described by Bressler and Seth (2011).

G-causality measures are usually computed in the multivariate autoregressive (MVAR) model framework defined by:

(1) $$\begin{array}{r} \text{X}\left(t\right)=\overset{p}{\underset{m=1}{\sum }}\text{A}\left(m\right)\text{X}\left(t-m\right)+\text{E}\left(t\right) \end{array}$$

where **X**(*t*) signal vector and **E**(*t*) white noise vector, both of size *k* (number of channels), **A**(*m*) model coefficients matrices, *p*—model order.

The transformation to the frequency domain yields:

(2) $$\begin{array}{ll} \text{X}\left(f\right)={\text{A}}^{-1}\left(f\right)\text{E}\left(f\right)=\text{H}\left(f\right)\text{E}\left(f\right)\; & \;\\ \text{H}\left(f\right)={\left(\overset{p}{\underset{m=0}{\sum }}\text{A}\left(m\right)\text{exp}\left(-2\pi imf\Delta t\right)\right)}^{-1}\; & \; \end{array}$$

where **H**(*f*) is a transfer matrix of the model.

To get a proper MVAR fit the number of data points must be larger (at least about an order of magnitude) than the number of model parameters: *kN*_*s*_ ≫ *pk*^2^ (*N*_*s*_ the number of data points in the window)r. This requires a compromise between *k* and *N*_*s*_. Alternatively, G-causality measures may be calculated by a non-parametric spectral method (Dhamala et al., 2008). However, spectral AR estimates have better statistical properties, since they are identical with these obtained by maximizing entropy of a process (Ulrych and Bishop, 1975). It means that AR estimate takes into account maximum of information contained in the signal and is maximally free of constraints. For MVAR fitting see Lütkepohl (2005).

Directed Transfer Function is defined in the form (Kamiński and Blinowska, 1991):

(3) $$\begin{array}{r} {\text{DTF}}_{j\to i}^{\text{2}}\left(f\right)=\frac{{\left|H_{ij}\left(f\right)\right|}^{2}}{\overset{k}{\underset{m=1}{\sum }}{\left|H_{im}\left(f\right)\right|}^{2}} \end{array}$$

where *H*_*ij*_ is an element of the transfer matrix of the MVAR model. DTF describes the causal influence of channel *j* on channel *i* at frequency *f*. The above normalized version of DTF takes values from 0 to 1, producing a ratio between the inflow from channel *j* to channel *i* in respect to all the inflows to channel *i*.

The non-normalized DTF:

(4) $$\begin{array}{r} {\text{NDTF}}_{ij}^{2}\left(f\right)={\left|H_{ij}\left(f\right)\right|}^{2} \end{array}$$

is directly related to the coupling strength between signals (Kamiński et al., 2001).

The direct Directed Transfer Function (dDTF) was introduced (Korzeniewska et al., 2003) to distinguish between indirect and direct flows:

(5) $$\begin{array}{r} {\text{dDTF}}_{ij}^{2}\left(f\right)=F_{ij}^{2}\left(f\right)C_{ij}^{2}\left(f\right)\\ F_{ij}^{2}\left(f\right)=\frac{{\left|H_{ij}\left(f\right)\right|}^{2}}{\underset{f}{\sum }\overset{k}{\underset{m=1}{\sum }}{\left|H_{im}\left(f\right)\right|}^{2}} \end{array}$$

where *C* is a partial coherence.

ffDTF is a modification of DTF where the denominator is integrated over frequencies, which makes it independent on frequency.

DC—directed coherence (Baccala et al., 1998) is a version of DTF that counteracts the effect of different noise variances in the input channels (*S* is the power spectrum, *V* is the noise variance):

(6) $$\begin{array}{r} \text{D}{\text{C}}_{ij}\left(f\right)=\frac{\sqrt{V_{jj}}H_{ij}\left(f\right)}{\sqrt{S_{ii}\left(f\right)}} \end{array}$$

Partial directed coherence (PDC) is defined as Baccala and Sameshima (2001):

(7) $$\begin{array}{r} \text{PD}{\text{C}}_{ij}\left(f\right)=\frac{A_{ij}\left(f\right)}{\sqrt{{\text{a}}_{j}^{*}\left(f\right){\text{a}}_{j}\left(f\right)}} \end{array}$$

where *A*_*ij*_(*f*) denotes an element of Fourier transformed MVAR coefficients **A**(*t*). The **a**_*j*_(*f*) denotes the *j*-th column of the matrix **A**(*f*), and an asterisk marks the operation of complex conjugation and transposition. PDC is normalized in the range [0,1]; its values correspond to the direct flows between channels of a process.

PDC operates in the frequency domain. However, its spectrum weakly depends on frequency and does not have a direct correspondence to the power spectra of the channels of a process. Unlike DTF, PDC value shows a ratio between transmission from channel *j* to channel *i* and the summarized outflow from channel *j*, so it tends to emphasize sinks rather than sources.

Considering the dependence of PDC on a signal's dynamic ranges, Baccala et al. (2007) introduced the generalized PDC (GPDC), which made the measure scale invariant. Schelter et al. (2009) pointed out that PDC is decreased when multiple signals are emitted from a given source, and that the measure does not allow conclusions on the absolute strength of the coupling. They proposed the so called re-normalized PDC, with a normalizing factor correcting the problem. Takahashi et al. (2010) introduced the information PDC (iPDC), which can be interpreted in terms of mutual information rate.

PDC found application e.g., in the study of epileptic seizures (Takahashi et al., 2007; Varotto et al., 2012), and GPDC was used for the analysis of directed connectivity from fMRI signals in language processing protocol (Sato et al., 2009).

Astolfi et al. (2007) compared DTF, PDC and dDTF in respect of connectivity pattern recognition and signal to noise ratio (SNR). It followed that DTF was the most robust to noise, but did not distinguish direct from indirect connections. This distinction was identified by dDTF and PDC.

Fasoula et al. (2013) compared different measures of G-causality including GC, PDC, GPDC, DTF, dDTF, and DC in respect of robustness to noise, spectral selectivity and the presence of a weak node (a channel which has much lower SNR than other channels). The results showed good robustness to noise of all measures, especially DTF, dDTF, PDC, and DC. However, these measures did not perform as well in the presence of a weak node as their modified counterparts: GPDC and DC. DTF performed well if the weak node was a passive sink and not the active node. PDC and GPDC were characterized by the poorest frequency selectivity.

## Time varying synchronization

The good spectral resolution of DTF and its robustness to noise makes it the proper measure for revealing synchronization between brain structures. Information processing in the brain involves short-time changes in electrical activity, and DTF is the only measure for which a time-varying version (SDTF) was developed and extensively used. In cases where there are multiple recordings of an experiment available, we may use the repetitions to effectively increase the statistical significance of estimates. In order to follow the dynamics we divide the data into shorter, presumably stationary, overlapping data windows (of length *N*_*S*_). Within each window the data covariance matrix *R*^(*r*)^ is calculated for every repetition separately (index (*r*) denotes repetition, *N*_*T*_ is the number of the repetitions), and then the resulting model is estimated based on the averaged matrix *R*:

(8) $$\begin{array}{r} R_{ij}\left(s\right)=\frac{1}{N_{T}}\overset{N_{T}}{\underset{r=1}{\sum }}R_{ij}^{\left(r\right)}\left(s\right)=\frac{1}{N_{T}}\overset{N_{T}}{\underset{r=1}{\sum }}\frac{1}{N_{S}}\overset{N_{S}}{\underset{t=1}{\sum }}X_{i}^{\left(r\right)}\left(t\right)X_{j}^{\left(r\right)}\left(t+s\right) \end{array}$$

The combined result from all windows—SDTF(*t, f*)—allows the dynamics of transmissions to be investigated (Ding et al., 2000).

Another possible solution for estimation of time-varying connectivity is an adaptive approach (Kalman filter, recursive least squares algorithm Hesse et al., 2003). A comparison of Kalman filtering with SDTF showed similar results in respect of detecting dynamics. However, computation time for the Kalman filter was more than an order of magnitude longer than for SDTF (Kamiński et al., 2010).

The effect of transients (event related potentials, ERPs) may disturb connectivity values when estimating time varying transmission. Subtraction of the ERP may be the solution (Kamiński et al., 2001), but it does not completely solve the problem since ERPs may differ between realizations. This problem was confronted e.g., by Wang et al. (2008), where a method separating the evoked response from ongoing activity on a trial-by-trial basis was used. Below we describe some applications of SDTF, illustrating its performance in time-frequency space and its topographic accuracy.

In an experiment concerning a motor task and its imagination (Ginter et al., 2001; Kuś et al., 2006), changes of propagation found by SDTF corresponded very well with the synchronization/desynchronization phenomena (Pfurtscheller and Lopes da Silva, 1999) in respect of topography and time-frequency characteristics. In the gamma band: movement was accompanied by a burst of gamma activity from C3 (overlying finger motor cortex—fPMC), and in the case of movement imagination there was cross talk in gamma between structures of the fPMC and the supplementary motor area (SMA).

**Figure 1:**
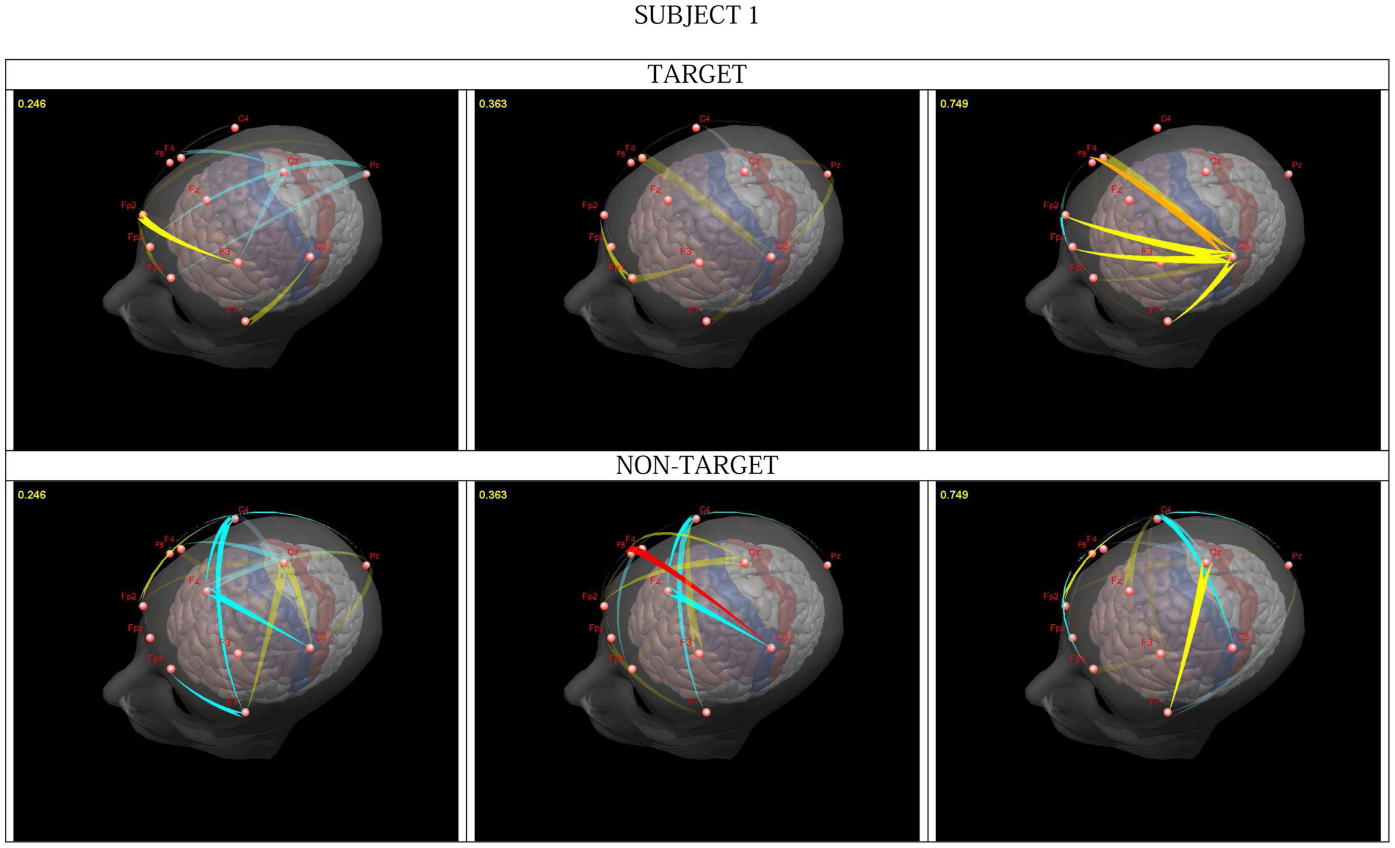
**Snapshots from a video presenting significant changes in transmissions in one subject, for target (upper) and non-target (lower part)**. Intensity of flow changes for increase: from pale yellow to red; for decrease: from light to dark blue. The time after cue presentation (in seconds) can be seen in the right upper corner. From Blinowska et al. (2010), with permission.

In the Continuous Attention Test different geometrical images were presented. The subject had to press a switch when two identical images (target condition) appeared and withhold the reaction for different images (non-target). We integrated flows (significantly differing from the resting state) in the 25–45 Hz frequency band and constructed animations representing dynamically changing propagation patterns (Figure 1; Blinowska et al., 2010). In the first epoch, which involved a mental comparison of the displayed images, the activity flow from the prefrontal cortex (PFC) and within the PFC was similar for all subjects for both conditions, in agreement with the role of this structure in focusing the attention and updating working memory (WM) (Romo et al., 1999; Smith and Jonides, 1999). In later epochs switch pressing for the target was accompanied by a propagation in the gamma band from C3 (underlying the fPMC). For the non-target conditions a transmission from F8 (located over the right inferior cortex—rIFC) to C3 or from Fz (located over the preSMA) to C3 was observed. Both structures are involved in “go/no go” tasks, exerting inhibition on the target structures (e.g., Aron et al., 2003; Burle et al., 2004). The observed transmission related to withholding the motor reaction confirmed the hypothesis put forward by Burle et al. (2004) i.e., that long-range cortico-cortical synchronization plays a role in the active inhibition of motor structures.

**Figure 2:**
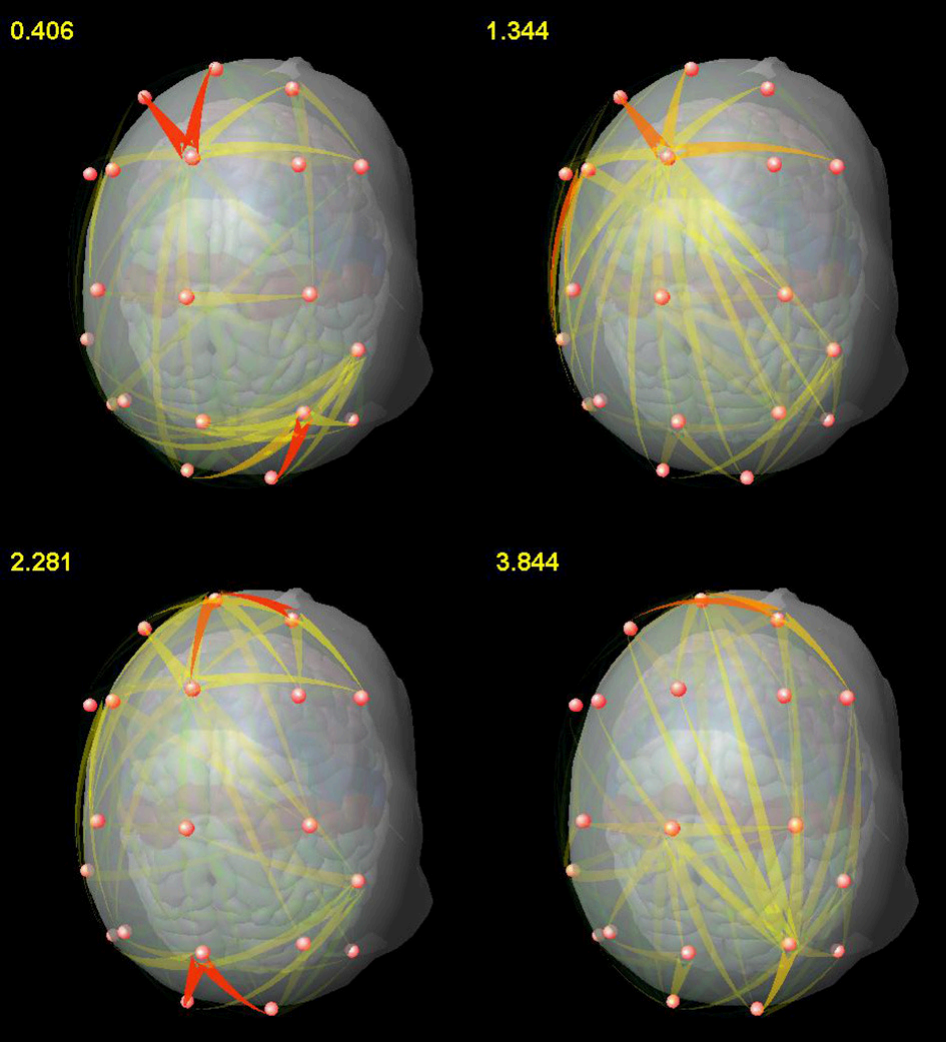
**Snapshots from a video showing the time-varying pattern of propagations for the representative subject**. The numbers in the upper left corner correspond to the time[s] after stimulus presentation. From Blinowska et al. (2013), with permission.

We also found, by means of SDTF, the main centers of EEG propagation in the frontal and parietal regions during a WM task involving memorization of relations (Blinowska et al., 2013), in agreement with imaging studies (Brzezicka et al., 2011) and neurophysiological hypotheses concerning the role of the fronto-parietal network (Fangmeier et al., 2006). The observed time evolution of propagation revealed a prevalence of short-range interactions, whereas the transmissions between tightly coupled centers of activity occurred only in certain moments as bursts of propagating activity (Figure 2). It shows that distant centers of information processing are linked by synchronization in lower frequencies (theta, alpha) when maintaining information, whereas higher rhythms (beta, gamma) are mostly responsible for information processing within these centers, in agreement with the postulated role of brain rhythms (Buzsaki, 2006).

Animations (Blinowski et al., 2014) of the three above experiments are available at http://brain.fuw.edu.pl/~kjbli.

Application of a network formalism based on assortative mixing (Newman, 2003) revealed the presence of a modular structure of brain networks, which made estimation of the coupling strength in specific frequency bands possible. The strength of interaction within the modules was higher than between the modules. Namely, the ratios of short-range to long-range interaction strengths varied from 1.40 for the theta band to 1.49 for the beta band. Interestingly, these ratios are close to those found for anatomical connections in cats—1.34 as reported by Latora and Marchiori (2003). The considerations concerning metabolic energy saving and efficient wiring in the brain (Changizi, 2006; Solé and Valverde, 2008) also indicate dense connectivity within modules and sparser connections between modules.

## Discussion and conclusions

Multivariate G-causality based measures provide a useful framework for establishing causal relations between neural populations. They have been successfully applied for finding interactions at subcortical and cortical levels GC measures have been used extensively for intracranial signals (e.g., Brovelli et al., 2004; Bressler et al., 2007; Bastos et al., 2015), while PDC has been applied to, for example, find directed connectivity in intracranial epileptic signals. DTF was used for localization of seizure onset from subdural electrodes (Franaszczuk et al., 1994) and SDTF for finding directed interaction between spike trains and LFP (Kocsis and Kaminski, 2006). Furthermore, a combination of SDTF and dDTF—SdDTF was applied in the investigation of dynamic patterns of electrocorticographic activity propagation during word repetition (Korzeniewska et al., 2008).

Application of G-causality measures to fMRI data is still controversial because of the low sampling rate, long delays of fMRI series in respect to neural activity, and the complex relation between neural activity and blood oxygenation level. The issue is currently under debate (e.g., Bressler and Seth, 2011; Seth et al., 2015). The controversies concerning application of G-causality for analysis of fMRI were also articulated in Roebroeck et al. (2011) and Friston (2011).

DTF and PDC have been widely used for identification of causal relations in EEG. The results of DTF concerning e.g., synchronization mechanisms in sleep (Kaminski et al., 1997), in transitive reasoning tasks (Brzezicka et al., 2010), and in affective states (Wyczesany et al., 2014) have demonstrated very good spectral and topographical agreement with known evidence and brought new information on the coupling between brain structures. The above described SDTF results have also shown excellent agreement with anatomical, physiological and neuroimaging evidence, additionally supplying information on the dynamics of synchronization and organization of neural networks.

In comparison with different methods of connectivity analysis, multivariate measures based on Granger principle provide information on causal frequency-specific coupling in neural assemblies, moreover they are robust in respect to noise and volume conduction. Additionally they offer possibilities to follow dynamical changes of interaction between brain structures. In summary, G-causality based measures provide a valuable tool for investigation of the large-scale neural synchronization and its dynamics.

## Author contributions

All of the authors, MK, AB, JK, and KB: contributed to the design of the work and interpretation of the data; took part in drafting the paper; critically revised the manuscript; approved the final version; agreed to be accountable for all aspects of the work.

## Funding

This material is based upon work supported by the National Science Centre in Poland under grants no. 2014/13/B/HS6/03155 and 2011/03/B/HS6/04458, Statutory Grant of Polish Ministry of Science and Higher Education to Faculty of Physics of University of Warsaw and Statutory Grant of Polish Ministry of Science and Higher Education to Institute of Biocybernetics and Biomedical Engineering.

### Conflict of interest statement

The authors declare that the research was conducted in the absence of any commercial or financial relationships that could be construed as a potential conflict of interest.
